# Visualization of elastin using cardiac magnetic resonance imaging after myocardial infarction as inflammatory response

**DOI:** 10.1038/s41598-021-90092-y

**Published:** 2021-05-26

**Authors:** Britta Elkenhans, Andrea Protti, Ajay Shah, David Onthank, René Botnar

**Affiliations:** 1 Department of Cardiology, Pneumology, and Angiology, University Hospital Aachen, Heinrich Heine University Duesseldorf, Moorenstr. 5, 40225 Duesseldorf, Germany; 2 Harvard Medical School, Department of Imaging, Lurie Family Imaging Center, Boston, USA; 3 Cardiovascular Division, King’s College London, London, UK; 4 Lantheus Medical Imaging, North Billerica, USA

**Keywords:** Biological techniques, Structural biology

## Abstract

The aim of this study was to investigate the merits of magnetic resonance imaging (MRI) using an elastin-binding contrast agent after myocardial infarction in mouse models with deletions of monocyte populations. Permanent ligation of the left anterior descending (LAD) artery was conducted in 10 wild-type mice and 10 each of three knockout models: CX3CR^−/−^, CCR2^−/−^, and MCP-1^−/−^. At 7 days and 30 days after permanent ligation, cardiac MRI was performed with a 7 T-Bruker horizontal scanner for in vivo detection of elastin with an elastin/tropoelastin-specific contrast agent (ESMA). Histology was performed with staining for elastin, collagen I and III, and F4/80. Real-time PCR was conducted to quantify the expression of genes for collagen I and III, F4/80, and tumor necrosis factor alpha (TNFα). Histological and ESMA-indicated elastin areas were strongly correlated (*r* = 0.8). 30 days after permanent ligation, CCR2-deficient mice demonstrated higher elastin levels in the scar relative to MCP-1^−/−^ (*p* < 0.04) and wild-type mice (*p* < 0.02). The ejection fraction was lower in CCR2-deficient mice. In vivo MRI in mouse models of MI can detect elastin deposition after myocardial infarction, highlighting the pivotal role of elastin in myocardial remodeling in mouse models with deletions of monocyte populations.

## Introduction

Myocardial infarction (MI) remains the leading cause of death in developed countries despite advances in medical and interventional treatment. Acute inflammation plays a pivotal role in wound repair dysfunction in the first weeks after myocardial infarction and can lead to heart failure if unresolved^1^.

In general, leucocytes predicts cardiovascular events as they orchestrate the inflammatory response in acute myocardial infarction^2^. There are two main monocyte-subpopulations, who are essentially involved in cardiac healing after myocardial infarction: They are named after their receptor-expression: Ly-6C^high^ (CCR2^high^ CX_3_CR_1_^low^) and Ly-6C^low^ (CCR2^low^ CX_3_CR_1_^high^). Normal cardiac remodeling post-MI involves a precise chronology in which monocytes are recruited to orchestrate angiogenesis and production of collagen and elastin. Monocyte chemoattractant protein-1 (MCP-1) and its receptor, CCR2, which is preferentially expressed on human and murine monocytes, are involved in the early inflammatory reaction after myocardial infarction. MCP-1 is released during the early phase of inflammation and recruits murine Ly-6C^high^ (CCR2^high^ CX_3_CR_1_^low^) monocytes, which remove extracellular debris. Seven days after myocardial infarction, Ly-6C^low^ (CCR2^low^ CX_3_CR_1_^high^) monocytes promote angiogenesis and the release of collagen and elastin with the aid of tumor necrosis factor alpha (TNFα), interleukin 1 beta (IL-1β), and interleukin 6 (IL-6)^3^. Lacking the MCP-1 signal and its downstream effects, MCP-KO mice exhibit decreased macrophage recruitment, delayed phagocytosis of dead cardiomyocytes, diminished fibroblast infiltration, and attenuated remodeling. CCR2-KO is also protective against the effects of adverse remodeling in mice^4^. Furthermore, elastin overexpression featured in post-MI remodeling provides elasticity to the extracellular matrix (ECM) and prevents cardiac dilation^5–8^. Thus, the elastin expression that results from monocyte recruitment represents an interesting imaging and therapeutic target^9^.

Several radiological imaging techniques exist to elucidate the role of elastin in cardiac remodeling. An elastin/tropoelastin-specific contrast agent (ESMA) and non-specific gadolinium-based contrast agent can be used to assess post-MI remodeling^3,10,11^. The use of ESMA to determine elastin deposition indicates a positive correlation between elastin signal and ejection fraction^10^.

We hypothesized that selective suppression of monocyte populations during post-MI remodeling affects the collagen and elastin composition of cardiac scars and that these differences in composition can be imaged by elastin-specific MRI and result in variable functional outcomes. Using MRI, real-time PCR (rtPCR), and histological techniques, we tested this hypothesis in wild-type and CCR2-, CX3CR1-, and MCP-1-knockout (KO) mice.

## Results

**Figure 1:**
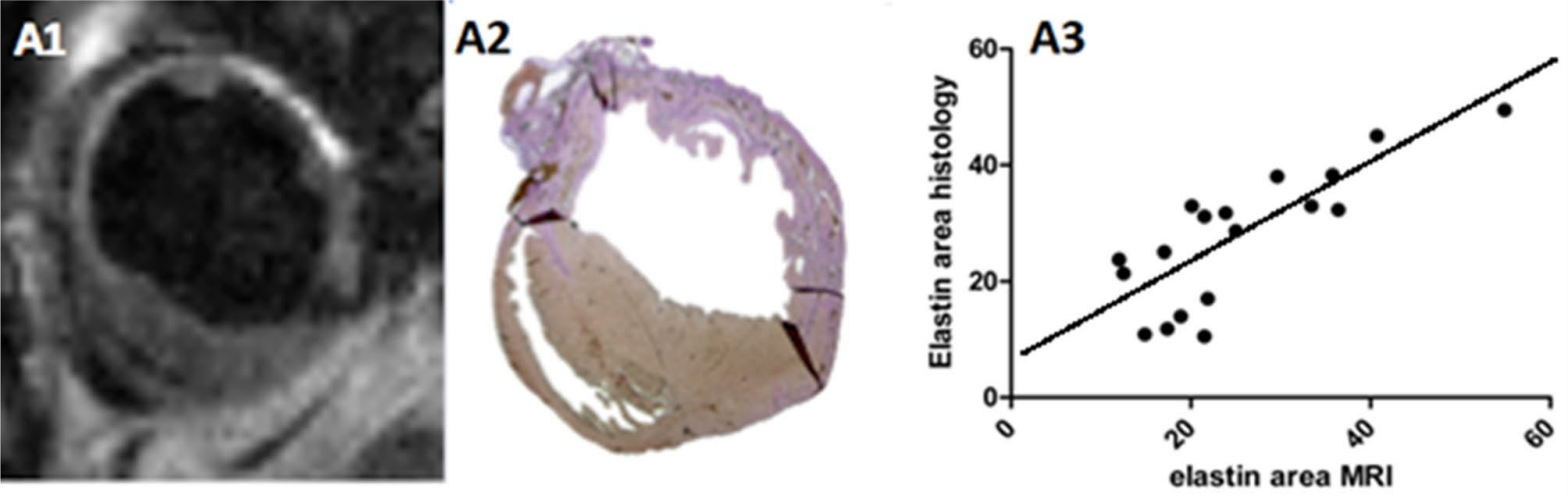
Elastin/tropoelastin–specific contrast agent (ESMA) cardiac MRI (A1) and Elastica van Giesson staining (A2) 7 days after permanent left anterior descending artery ligation in wild-type mice. Correlation of cardiac MRI and histology among all groups 7 days after permanent ligation (A3) (95% confidence interval).

Elastin expression in the scar was detected in all groups 7 days and 30 days after permanent ligation. Seven days after permanent ligation, gadolinium uptake on ESMA-MRI correlated with histological elastin staining of the infarcted area (*r* = 0.8, *p* < 0.0001) (Fig. 1).

**Figure 2:**
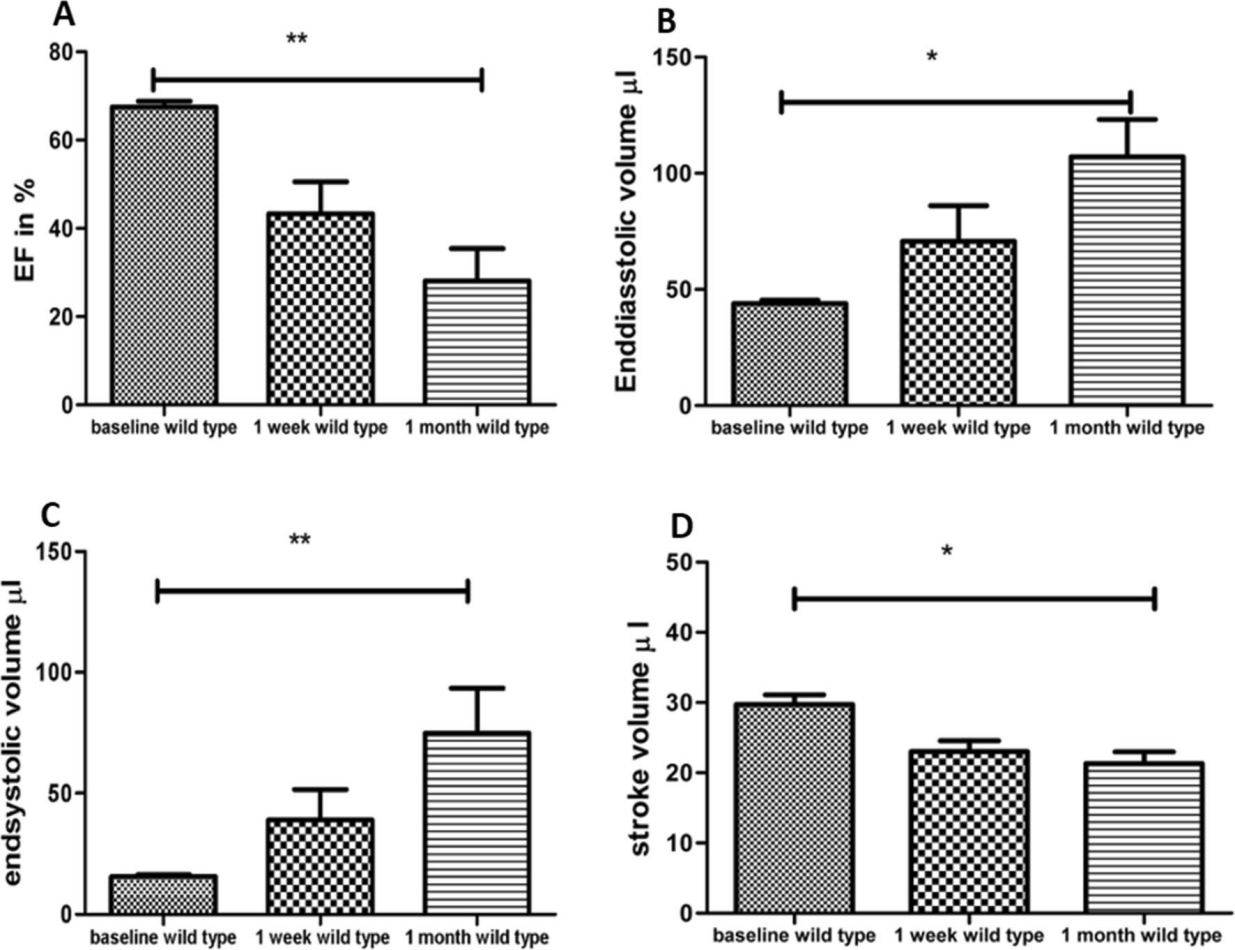
In wild type mice, the ejection fraction was significantly lower 30 days post MI than at baseline (**A**). End-diastolic volume (**B**), end-systolic volume were increased (**C**), and stroke volume were decreased (**D**) in wild-type mice, prior to (baseline), 7 days, and 30 days after permanent ligation.

Significant differences in ejection fraction, end-diastolic, and end-systolic volumes were found in wild-type mice between baseline and 30 days after permanent ligation (*p* < 0.001, *p* < 0.02, *p* < 0.01) (Fig. 2A–C). Ejection fraction and stroke volume were significantly lower 30 days after MI (Fig. 2A, *p* < 0.01, Fig. 2D, *p* < 0.02). End-diastolic and end-systolic volumes were increased 30 days after permanent ligation in wild-type mice in contrast to baseline, pre-MI measurements.

CCR2-KO mice had the largest infarcted areas 30 days after permanent ligation and poor functional recovery. CX3CR1-KO mice exhibited sufficient functional recovery by day 30, and the best functional recovery of all strains was observed MCP-1-KO mice, although their recovery was not significantly different than the other mouse lines (data not shown).

**Figure 3:**
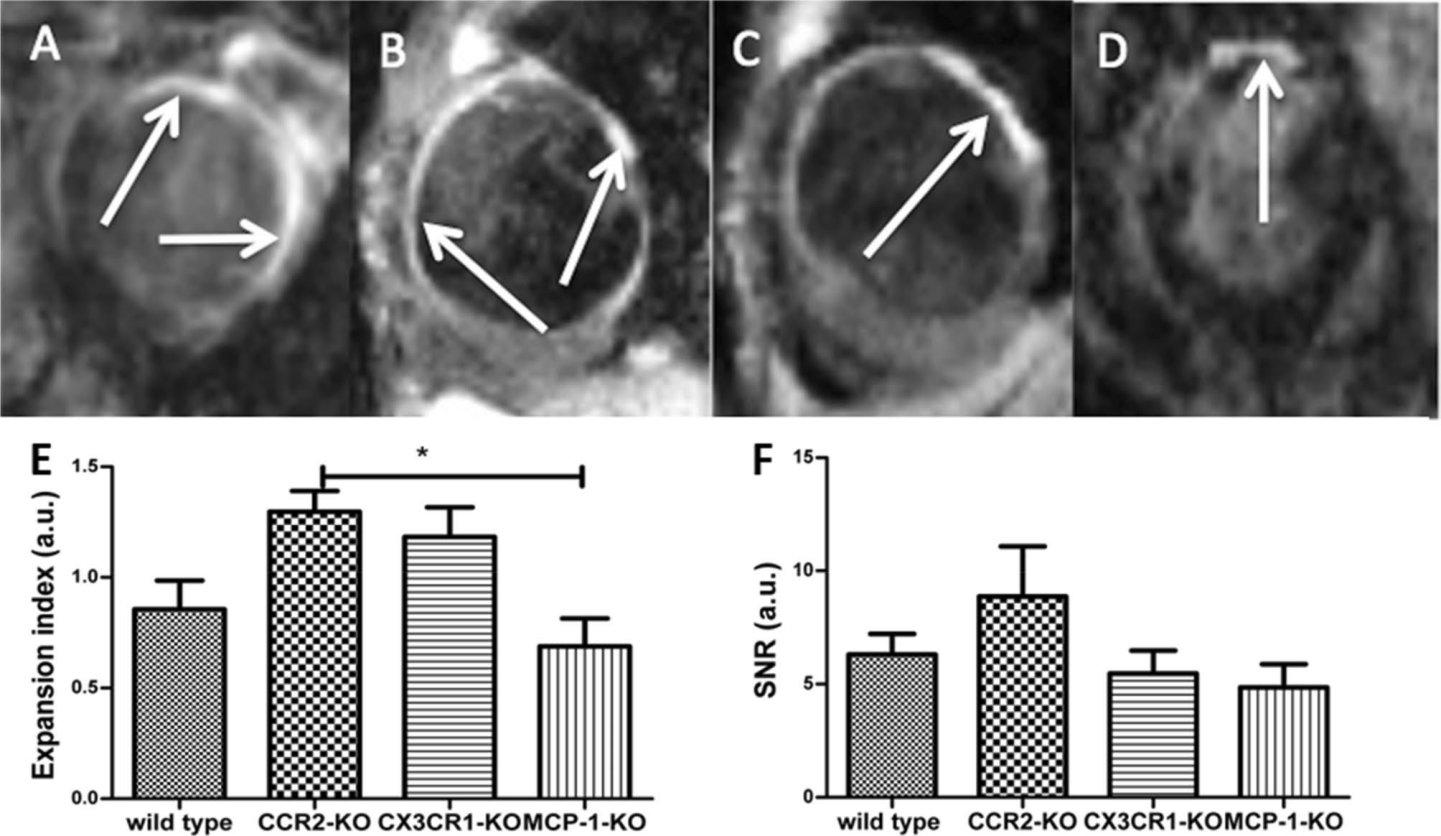
In-vivo follow up of elastin with the aid of cardiac MRI in wild-type mice 30 days after permanent ligation (**A**). The arrows represent ESMA uptake in exemplary images. Cardiac MRI image of CCR2-KO mice 30 days after permanent ligation (**B**). Cardiac MRI 30 days after permanent ligation in CX3CR1-KO mice (**C**). Cardiac MRI 30 days after permanent ligation in MCP-1-KO mice (**D**), (**E**) shows increased expansion index in CCR2-Ko mice compared to MCP-1-KO mice 30 days after MI as sign for increased myocardial scar with decreased ability of contraction. In (**F**) SNR was increased in CCR2-KO mice compared to MCP1-KO-mice 30 days after MI, although statistically not relevant also as a sign for extended myocardial scar.

Thirty days after permanent ligation, the largest areas of hyperenhancement after administration of ESMA were detected in CCR2-KO mice (Fig. 3B). Consistent with these findings, increased SNR levels were measured in CCR2-KO mice (Fig. 3E). In addition, the expansion index^12^ was significantly greater in CCR2-KO than MCP-1 KO mice (Fig. 3F; *p* < 0.03).

**Figure 4:**
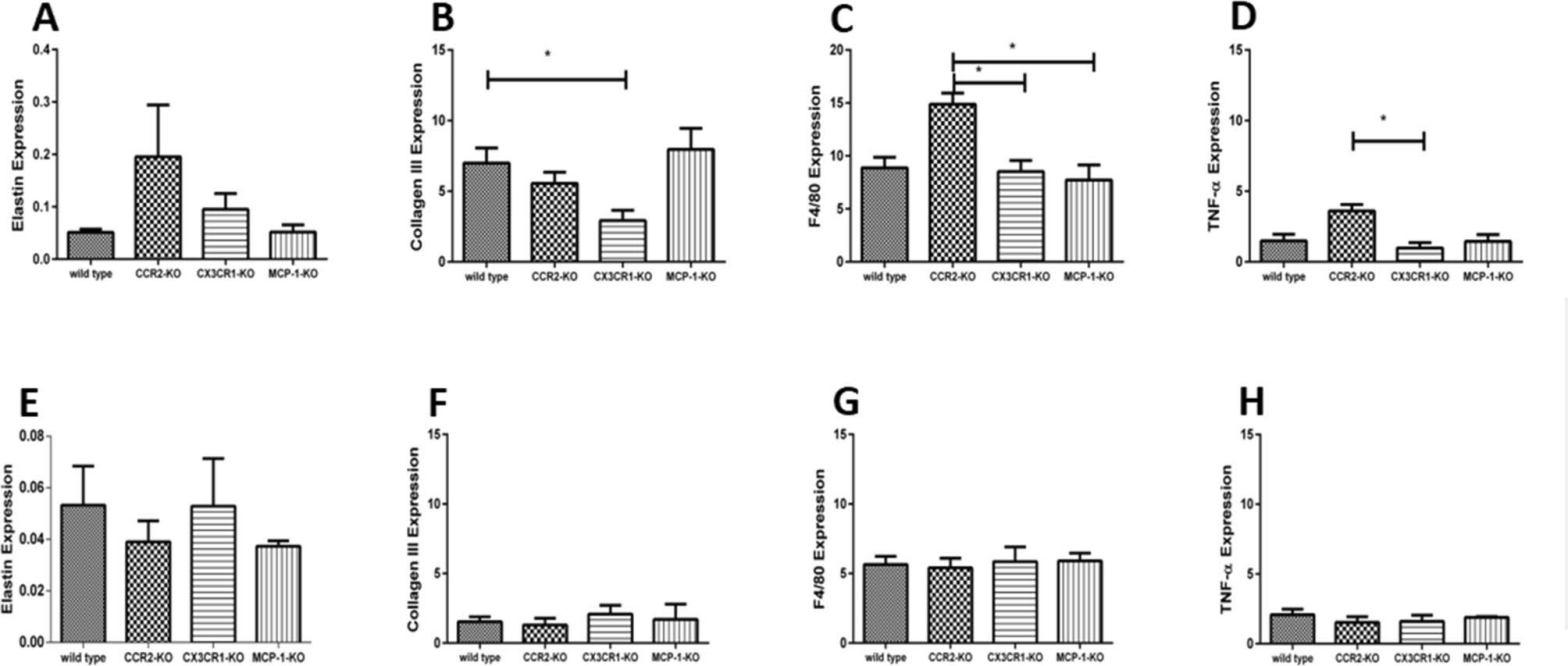
Representation of cardiac remodeling with the aid of gene expression of relevant markers such as elastin 7 days (**A**) and 30 days (**E**) after permanent ligation. Expression of collagen III 7 days (**B**) and 30 days (**F**) after permanent ligation. In (**B**) collagen III-expression was significantly increased in wild-type mice compared to CX3CR1-KO mice, which shows early remodeling in wild type mice, but no statistically relevant difference in functional outcome, (**C**) represents increased monocyte-expression (F4/80) in CCR2-KO-mice compared to CX3CR1-KO mice and to MCP-1-KO mice 7 days, (**G**) shows monocyte-expression 30 days after MI. In (**D**) an increased TNF-alpha gene expression was seen in CCR2-KO mice compared to CX3CR1-KO mice, (**H**) represents TNF-alpha gene expression 30 days after MI.

Knock-out groups differed in their expression of cardiogenesis genes post-MI, but, across all groups, collagen III, F4/80, elastin, and TNFα expression decreased between 7 and 30 days post-MI, at which point no significant differences among groups were apparent (Fig. 4). Seven days post-MI, collagen III expression was significantly lower in CX3CR1-KO mice relative to wild-type mice (Fig. 4B; *p* < 0.02), F4/80 was significantly higher in CCR2-KO mice compared to CX3CR1- and MCP-1-KO mice (Fig. 4C; *p* < 0.01), elastin gene expression was highest in CCR2-KO animals (Fig. 4A), and TNFα expression was higher in CCR2-KO mice than CX3CR1-KO mice (Fig. 4D; *p* < 0.01).

**Figure 5:**
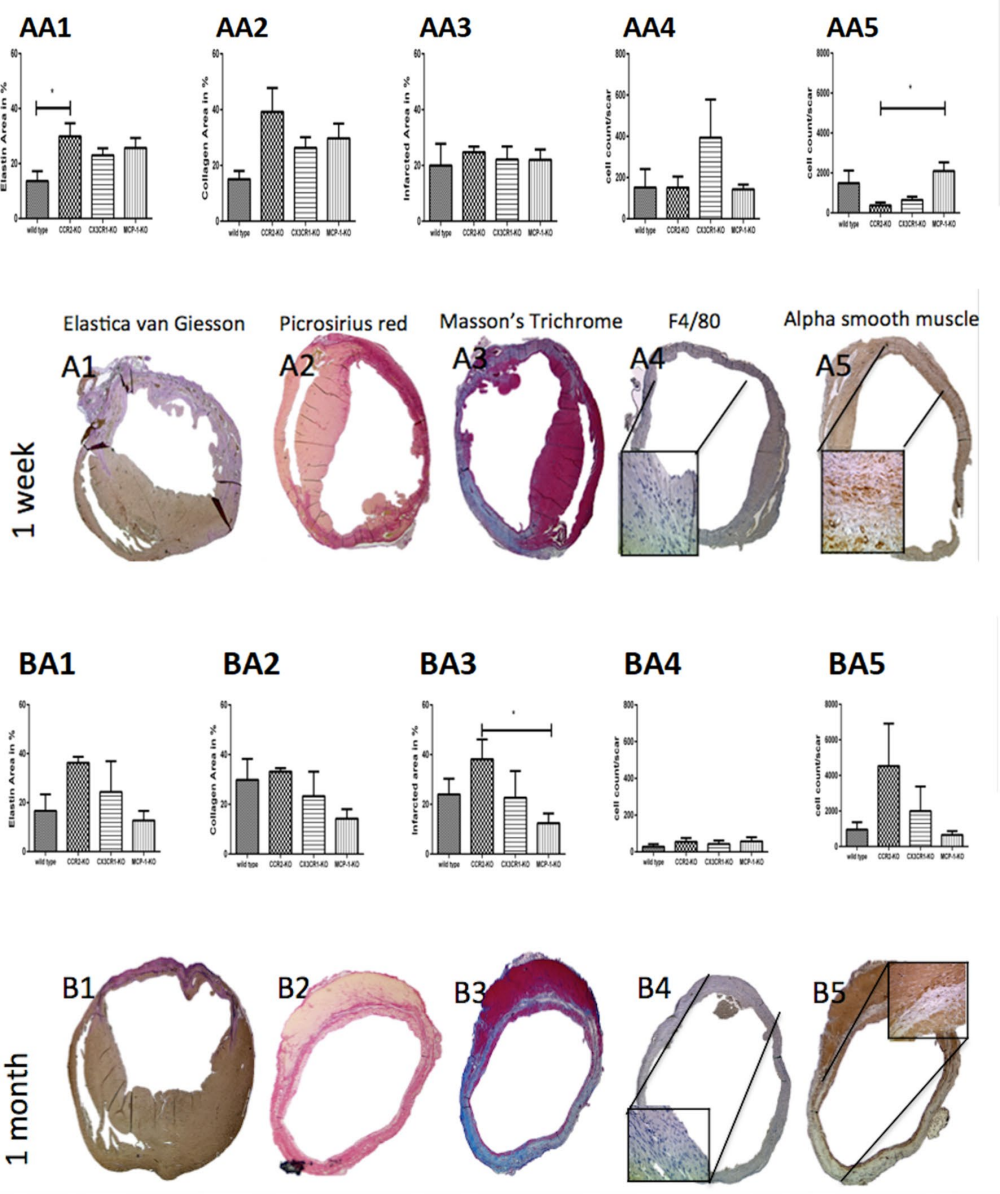
Cardiac remodeling in a histological follow-up regarding the scar-components elastin, collagen, monocyte-occurrence and blood supply. Figure AA1 shows the Graph of elastin occurrence 7 days after permanent ligation in wild type mice, CCR2-KO mice, CX3CR1and MCP1-KO mice. Figure A1 exhibits its corresponding exemplary histological image with Elastica-van Giesson-staining. Figure AA2 represents the graph of collagen deposit 7 days after permanent ligation in wild type mice, CCR2-KO mice, CX3CR1 and MCP1-KO mice and figure A2 represents the corresponding exemplary histological image with Picrosirius red staining. Figure AA3 indicates the graph of scar formation 7 days after permanent ligation in wild type mice, CCR2-KO mice, CX3CR1 and MCP1-KO mice and figure A3 exhibits the corresponding exemplary histological image with the aid of Masson’s Trichrome staining. Figure AA4 exhibits inflammation in graphic formation 7 days after permanent ligation in wild type mice, CCR2-KO mice, CX3CR1 and MCP1-KO mice and figure A4 its corresponding exemplary histological image with F4/80 staining. Figure AA5 shows the occurrence of new vessel formation in graphical order 7 days after permanent ligation in wild type mice, CCR2-KO mice, CX3CR1 and MCP1-KO mice and A5 its corresponding exemplary histological image with alpha smooth vessel staining. Figure BA1 points out the Graph of elastin occurrence 30 days after permanent ligation in wild type mice, CCR2-KO mice, CX3CR1 and MCP1-KO mice. Figure B1 exhibits its corresponding exemplary histological image with the aid of Elastica-van-Giesson staining. Figure BA2 represents the graph of collagen deposit 30 days after permanent ligation in wild type mice, CCR2-KO mice, CX3CR1 and MCP1-KO mice and figure B2 represents the corresponding exemplary histological image with Picrosirius red staining. Figure BA3 indicates the graph of scar formation 30 days after permanent ligation in wild type mice, CCR2-KO mice, CX3CR1and MCP1-KO mice and figure B3 exhibits the corresponding exemplary histological image with the aid of Masson’s Trichrome staining. Figure BA4 exhibits inflammation in graphic formation 30 days after permanent ligation in wild type mice, CCR2-KO mice, CX3CR1and MCP1-KO mice and figure B4 its corresponding exemplary histological image with F4/80 staining. Figure BA5 shows the occurrence of new vessel formation in graphical order 30 days after permanent ligation in wild type mice, CCR2-KO mice, CX3CR1and MCP1-KO mice and B5 its corresponding exemplary histological image with alpha smooth vessel staining.

Elastin-staining areas were larger in CCR2-KO mice relative to wild-type mice after 7 days (Fig. 5 AA1; *p* < 0.02). In addition, 30 days after permanent ligation, CCR2-KO mice had significantly larger infarcted areas relative to MCP-1-KO mice (Fig. 5 BA3; *p* < 0.04). Seven days after permanent ligation, CX3CR1-KO mice tended to have more F4/80-positive cells than wild-type mice (Fig. 5 AA4), but this tendency did not rise to the level of statistical significance. Seven days after permanent ligation, myofibroblasts were more abundant in MCP-1-KO mice than CCR2-KO mice (Fig. 5 AA5; *p* < 0.0215).

Compared to wild type mice, MCP-1-KO mice exhibited smaller infarcted areas with low elastin content and smaller volumes at day 7 and 30 days after myocardial infarction with a good ejection fraction during both time-points.

They were followed by CX3CR-KO mice, who also featured small infarcted areas with low elastin content, small volumes and good ejection fractions in both time points.

The poorest outcome showed CCR2-KO mice with large infarcted areas already 7 days after myocardial infarcted with a decreased ejection fraction, large volumes and a large amount of elastin. 30 days after myocardial infarction, the ejection fraction was still low in CCR2-KO mice compared to wild type mice with an increased elastin-amount and increased volumes (data not shown).

## Discussion

We compared three different knockout mouse lines to wild-type mice to investigate inflammatory reactions and scar development after myocardial infarction with cardiac MRI. In our study, elastin played a major role in cardiac fibrosis. We used four independent methods for analysis of elastin synthesis after permanent ligation in wild-type, CCR2-, CX3CR1-, and MCP-1-KO mice. This study demonstrates the feasibility of in vivo monitoring of elastin formation after experimental myocardial infarction in knockout mouse models of selective monocyte suppression with an elastin binding contrast-agent.

In human and animal models of myocardial infarction, MCP-1 is upregulated^13–16^. MCP-1^−/−^ mice have infarcts with decreased expression of the pro-inflammatory cytokines TNFα, IL-1β, and IL-6 and attenuated left ventricular remodeling with defective monocyte differentiation^4,17,18^ but, in humans, increased levels of MCP-1 are associated with an increased risk for death or myocardial infarction^19,20^. In our study, infarct sizes, elastin deposition, and elastin areas were significantly smaller in MCP-1-KO than CCR2-KO mice 30 days post-MI and elastin was inversely related to MCP-1 expression during the 30-day recovery. Although higher numbers of myofibroblasts were documented in MCP-KO mice than CCR2-KO mice 7 days post-MI, these small vessels were no longer visible 30 days after permanent ligation, and, thus, are unlikely to increase blood supply.

CCR2^high^ CX_3_CR_1_^low^ monocytes are potently inflammatory and mainly up-regulated during the first days after myocardial infarction^21–23^. However, in our study, CCR2-KO mice exhibited significantly more F4/80-positive cells in the infarcted area than either CX3CR1- or MCP-1-KO mice and elevated TNF alpha expression relative to CX3CR1-KO mice 7 days post-MI. Despite this pro-inflammatory milieu, CCR2-KO exhibited increased areas of elastin 7 days and 30 days post-MI and increased levels of elastin expression 7 days post-MI, albeit with a larger infarcted area. Others have shown that, functionally, CCR2-receptor deficiency results in a diminished ejection fraction after myocardial infarction during long-term recovery compared to wild-type, CX3CR1- and MCP-1 KO mice^24^ and we found that hyperenhancement and expansion index were greatest in CCR2-KO mice 30 days post-MI. Finally, consistent with Ortlepp et al.^25^ we observed that CCR2-deficient mice developed cardiac fibrosis leading to heart failure.

In contrast, Ly-6C^low^ (CCR2^low^ CX_3_CR_1_^high^) monocytes are up-regulated during later stages of the inflammatory response in the murine model of MI and are thought to play an active role in infarct healing^3^. In our studies, 7 days after permanent ligation, CX3CR1-KO mice showed low levels of collagen III gene expression. During long term follow-up, low collagen III levels did not necessarily lead to heart failure because, although CX3CR1 receptors play a role in the acutely inflammatory monocyte subpopulation, they do not cause long-term harm to cardiomyocytes^26^. Indeed, in our study, we found that F4/80-positive cells were increased in CX3CR1-KO mice as a result of an acute inflammatory reaction 7 days post-MI; however, we did not observe long-term differences in scar formation among any of the mouse groups.

Scar tissue occurs when myofibroblasts deposit ECM proteins. Ly-6C^low^ monocytes cause the transdifferentiation of migrated fibroblasts into myofibroblasts. Activated myofibroblasts release their internal storage of collagen I, collagen III, and elastin, and these ECM compounds create fibrotic scar tissue after myocardial infarction^27^. Cyclic mechanical stretch increases collagen I and III expression, as well as that of other ECM proteins, including proteoglycans, elastin, cytokines, and growth factors^28,29^.

Elastin stabilizes cardiac fibrosis and preserves left ventricular function after myocardial infarction^30^ and overexpression attenuates scar expansion in general during long term observation^8^. Silencing (at least for one week) of CCR2 in ischemia/reperfusion reduces myocardial infarct size^31,32^. In unreperfused MI, experimental data report both negative and positive correlations between monocyte/macrophage numbers and healing/left ventricular remodeling^33^. However, in our study, early elastin expression lead to extended scar formation 30 days post-MI, reduced left ventricular function, and increased expansion index in CCR2-KO mice. Long-term follow-up should be the consequence.

In this study, we have demonstrated the feasibility of in vivo monitoring of elastin formation after myocardial infarction in mouse models of selective monocyte suppression using an elastin-specific MR contrast agent, ESMA. Differences in cardiac function and myocardial recovery highlight the important role of de novo elastin synthesis in myocardial remodeling. These promising results warrant further investigations in larger animal models and humans. Elastin thus seems to play an important role in cardiac fibrosis and may be used as a new biomarker for in vivo follow-up of cardiac fibrosis with ESMA in cardiac MRI.

## Methods

The study was carried out in compliance with the ARRIVE guidelines. Further, the study was conducted according to the guidelines and regulations of the King’s College London.

All experimental protocols were approved by the licensing committee of the King’s College London.

### Study design

MI was induced as previously described in an established mouse model of ischemia^34^ using 40 (n = 10 per knockout and per methodology) 10-week-old female wild-type (Charles River, Manston Road, Margate, Kent CT9 4LT UK), CCR2- and CX3CR1-KO (kindly provided by Univ.-Prof. Dr. F. Tacke et al.), and MCP-1-KO mice (Jackson Laboratory, 600 Main Street, Bar Harbor, Maine 04609 USA). All groups were matched for infarct size in the following mentioned methodologies.

**Figure 6:**
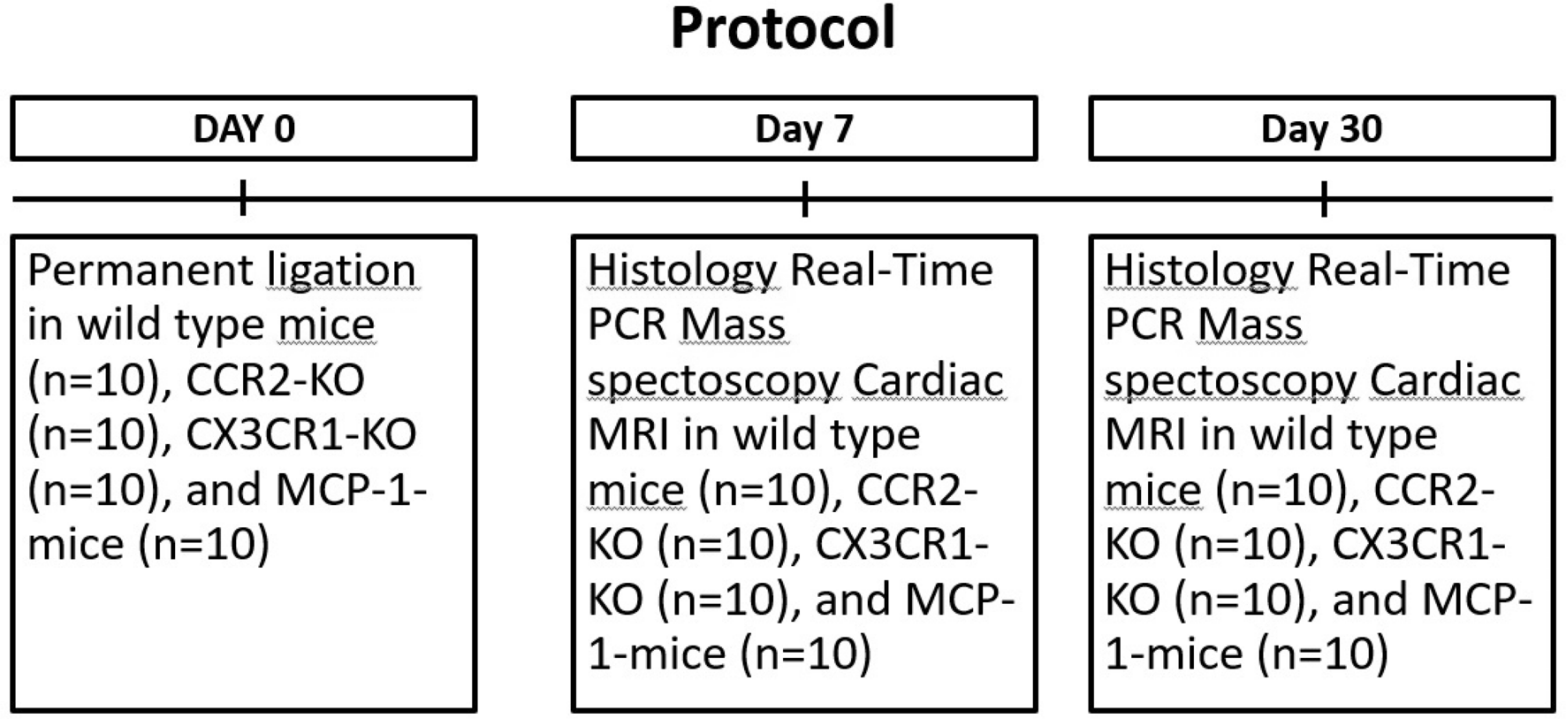
Project timeline.

In brief, animals were anesthetized with ketamine hydrochloride (Pfizer) and Dormitore (medetomidine hydrochloride, Richter Pharma AG) and underwent endotracheal intubation. They were ventilated using a dedicated small animal ventilator (Hugo Sachs Elektronik, Germany). A lateral thoracotomy was performed; chest wall muscles were incised and reflected, and the thorax was opened at the fourth intercostal space. The pericardium was removed to access the epicardial surface. The left coronary artery was ligated using an 8/0 suture 1–2 mm below the tip of the left atrium. Successful ligation was confirmed by regional blanching of the left ventricle extending to the apex. The chest wall was repaired in layers and the animals weaned from the respirator. The animals recovered in a warmed chamber (28 °C) for at least 6 h. We used perioperative analgesia with intramuscular Temgesic (buprenorphine, Bayer) and subcutaneous Baytril (Flunixin, Bayer, Leverkusen, Germany). Seven and 30 days after permanent ligation, ESMA MRI was performed, as was histological assessment (Fig. 6).

### Cardiac MRI

All mice were scanned in a prone position using a 7-T Bruker horizontal MR scanner (Biospec 7T) with a quadrature transmit/receive coil (RAPID, Biomedical, Germany)^35^ 7 days and 30 days post-MI. We used cine-FLASH sequences at 7T for the visualization of myocardial infarction, which are superior to the conventional inversion recovery turbo FLASH sequence, because they are moving pictures, which allows the investigation of ejection fraction^35^. After initial scout scans to identify the two- and four-chamber views, T1-weighted cine-FLASH MRI was performed in the short axis view with ECG triggering at the peak of the QRS complex to investigate functional, volumetric parameters (ejection fraction, end-diastolic volume, end-systolic volume, and stroke volume). Imaging parameters included TR = 8 to 10 ms, TReff = RR-interval, TE = 1 ms, FOV = 25 × 25 mm^2^, matrix size = 128 × 128 mm,, slice thickness = 1 mm; flip angle = 40°, 3 averages, 9 slices, 1 k-space line/frame, and 10–14 frames per cardiac cycle. The acquisition time was 8 ± 0.5 min.

Subsequently, ESMA-MRI was performed in the short axis view with an inversion recovery (Look-Locker) sequence^11^. Infarct size and remodeling were assessed two hours after injection of ESMA (0.2 mmol/kg), a time point that is optimal for infarct visualization due to almost complete blood clearance^10^. Imaging parameters included FOV = 25 × 25 mm^2^, slice thickness = 1 mm, 30 phases, matrix size = 128 × 128 mm, 1 slice, flip angle = 10°, IR = 2500 ms, cardiac cycle ≈ 120 ms, acquisition time ≈ 13 min (Fig. 3). Signal-to-noise ratio (SNR), which defines the efficacy of the FLASH-sequence, was recorded. Following ESMA-MRI at day 7 and 30, we calculated the expansion index, which defines the ratio of infarcted to non-infarcted endocardial segment length.

### Analysis of elastin and collagen with rtPCR

At 7 days and 30 days after permanent ligation, tissue was harvested (n = 10 per KO group per timepoint) for the analysis of elastin and collagen expression in real-time PCR (rtPCR). Primers were designed with the aid of the Universal Probe Library for Mouse (Roche, Burgess Hill, West Sussex, UK) for the quantification of elastin (Sequence AGATTCAGCCTAGGAGGCTTGCAAGTCTGGCCTTTCGTAATTGCCCCCTCCCCCGCGGCCCCCTCCCCCAGCTCCCCCTCCTCCCGCCCT) and collagen I (Sequence TTTTGTAATAC GACTCACTATAGGGCGGCCGGGAATTCGTCGACTGGATCCGGTACCGAGGAGATCTGCCGCCGCGATCGCC) and III (Sequence TTTTGTAATACGACTCACTATAGGGC GGCCGGGAATTCGTCGACTGGATCCGGTACCGAGGAGATCTGCCGCCGCGATCGCC) expression. Total RNA was extracted from the scar and remote myocardium at 7 and 30 days post-MI. Tissue was harvested, treated with TRIzol (Thermo Fisher Scientific, Waltham, Massachusetts, US) and dispersed using an RNAse-free stick pestle kit (Anachem, Bedfordshire, UK). Tissue was then treated using the RNeasy Plus Universal Mini Kit (Qiagen, Crawley, West Sussex, UK) according to the manufacturer’s protocol. Reverse transcription was performed with MultiScribe reverse transcriptase enzyme (PE Applied Biosystems, Foster City, California, US). To quantify the levels of messenger RNA (mRNA), we normalized expression of the target genes to 18S ribosomal RNA, and data were expressed as relative-fold differences between complementary DNA of the study sample and a calibrated sample^36^ (Fig. 4).

### Histological analysis

Seven days and 30 days after permanent ligation, mouse hearts were harvested, fixed in 4% formalin, embedded in paraffin, and sectioned into 6-µm slices. Sections were stained with Elastica-van-Giesson (Sigma-Aldrich, Munich, Germany) for the visualization of elastin, picrosirius red (Abcam, Cambridge, UK) for collagen I and III, F4/80 stain (Abcam, Cambridge, UK) for monocyte visualization and quantification, Masson’s trichrome (Sigma Aldrich, Munich, Germany) for the detection of fibrosis as a proxy for myocardial infarction, and alpha-smooth muscle actin (Abcam, Cambridge, UK) for quantification of myofibroblasts in the scar. All staining was performed according to the manufacturers’ protocols (Fig. 5).

### Analysis of elastin-binding contrast agent with ICP-MS

Mass spectroscopy was conducted to assess infarct size by quantifying gadolinium in the scar. ICP-MS was performed on scar tissue 7 days and 30 days after permanent ligation. The model ELAN DRCplus (Perkin Elmer, Waltham, Massachusetts, US) was used with the software package Perkin Elmer’s Elan v. 3.3. Principal settings were plasma, auxiliary, and nebulizer gas flows 15, 0.8, and 0.97 l/min of argon with an RF power of 1200 W. The sample flow rate was 0.4 ml/min. The residence times on gadolinium were averaged over five replicate readings (data not shown).

### Image analysis

Semi-quantitative estimation of ESMA in the infarct zone was performed based on signal intensity (SI), which provides a linear relationship between SI and tissue T1 up to a user-defined T1 cut-off value. This method has been validated in previous studies by quantifying tissue gadolinium concentration with mass spectroscopy^11^. MRI and histology images were analyzed using ImageJ (version 1.45). The surface areas of myocardial infarction, as indicated by MRI and histology, were correlated to determine the reliability of ESMA in detecting cardiac remodeling (Fig. 1).

(S_Blood_) and reference tissue (S_Myocardium_) in the left ventricle was defined. Noise (N) was estimated by the standard deviation in both of these respective ROIs. CNR (contrast to noise) was defined by using following equation: CNR = (S_Blood_-S_Myocardium_)/(0.5x(N_Blood_ + N_Myocardium_)).

SNR (signal to noise) was defined by using the equation: SNR = (S_Blood_)/(N_Blood_)^35,37^.

### Statistical analysis

Data were statistically analyzed using one-way ANOVA (assumed value MI extent variability SS calculation 96), Student’s t-test, and the correlation model used in Graph Pad Prism (version 5). Sample size in each group was selected based on potential variability in extent of MI. Ten animals yielded a power of 80% to detect differences between the groups when statistical significance is indicated by *p* < 0.05. The homoscedasticity and sphericity of the data were revised.
